# Comparison of Bacterial and Dye Microleakage of Different Root-End Filling Materials

**Published:** 2010-02-20

**Authors:** Majid Kazem, Mohammad Jafar Eghbal, Saeed Asgary

**Affiliations:** 1. Department of Endodontics, Dental Research Center, Shahid Beheshti University of Medical Sciences, Tehran, Iran.; 2. Department of Endodontics, Iranian Center for Endodontic Research, Dental Research Center/Dental School, Shahid Beheshti University of Medical Sciences, Tehran, Iran.

**Keywords:** Bacterial Leakage, Dye Leakage, MTA, Retrofill Material

## Abstract

**INTRODUCTION:**

The provision of an effective apical seal is an essential factor when choosing an appropriate material for success of root-end surgeries. Root-end resection, preparation and obturation should provide an adequate apical seal. The aim of this in vitro study was to investigate bacterial and dye microleakage of four different root-end filling materials and compare the efficacy of these two methods.

**MATERIALS AND METHODS:**

Fifty-six single-rooted teeth with intact and straight roots were randomly assigned into four study groups of 12 each and two control groups of three each. After decoronation, root canals were prepared up to file size #40 using step back technique; then, they were filled with gutta-percha and AH26 sealer. Root-ends were resected 3 mm above the root-end and 3 mm deep cavities were prepared. Root-end cavities were filled with amalgam, Root Mineral Trioxide Aggregate (Root MTA), White ProRoot MTA (WMTA), and calcium enriched mixture (CEM) cement. Bacterial leakage was investigated in Trypticase Soy Broth (containing Enterococcus faecalis) after 70 days and 1% methylene blue dye leakage was assessed after 72 hours. Complete dye leakage was checked using stereomicroscope (×40). Data were statistically analyzed using Fisher Exact test. For pair comparison between the two methods Kapa agreement was utilized.

**RESULTS:**

After 70 days there was 100% bacterial leakage in amalgam, and CEM cement, 91.7% in WMTA, and 75% in Root MTA. This difference was not significantly different. The difference in complete dye leakage was also not significant (WMTA and CEM cement 16.7%, Amalgam and Root MTA 33.3%).

**CONCLUSION:**

There was no significant measure of agreement between dye and bacterial penetration along root-end fillings. CEM cement was not significantly different from currently used retrofilling materials e.g. WMTA.

## INTRODUCTION

Bacteria play an important role in creating and extending apical lesions [1][2][3]. Microorganisms and their by-products can also penetrate into filled root canal systems and destroy periradicular tissues [4][5]. The main purpose of nonsurgical root canal treatment is to remove irritants and obturate/seal the root canal system. Periradicular surgeries are indicated when these treatments fail or are impossible to perform. Mineral Trioxide Aggregate (MTA) was introduced by Torabinejad for sealing the internal and external root space [6]. MTA provides an effective seal against dye and bacterial penetration [7]. Root MTA, which was introduced by Lotfi, shows some comparable properties to ProRoot MTA [8]. Recently, new material named calcium enriched mixture (CEM) cement has been introduced to dentistry. It has different characteristics to MTA and Portland cement [9] as well as good biocompatibility [10]. Bacterial leakage measurements assess the sealing ability of all portions, while in dye leakage only the walls adjacent to material are investigated. Bacterial leakage studies focus on the main pathogenic factor in periapical diseases i.e. the presence of microorganisms. E. faecalis was used in this study as it is widely believed that it is the predominant bacteria in root canal failures and chronic apical periodontitis [11][12]. The exact factors which induce apical periodontitis such as the specific bacteria and/or byproducts are unclear. [13]. Kersten showed that dye particles with smaller size penetrate more than others [14]. We can therefore assume that dye particles smaller than bacteria penetrate more than bacteria; but dye leakage studies have shown that full dye penetration does not occur after 1-2 days or even two weeks. It has been also shown that type of dye, centrifusion, and vacancy does not significantly change the dye penetration [15][16][17][18][19][20][21][22][23][24]; this maybe due to air bubbles which prevent passive dye penetration [25].

The aim of this in vitro study was to investigate bacterial and dye microleakage of four different root-end filling materials and compare the efficacy and agreement of these two methods.

## MATERIALS AND METHODS

In this in vitro experimental study, 54 single-rooted and single-canalled teeth were randomly divided into four study groups of 12 each and two control groups of three each. Teeth surfaces were cleaned with curette and stored in NaOCl 5.25% for 1 hour. Subsequently, they were rinsed with and stored in normal saline.

All studied teeth were decoronated above the CEJ level so that remaining sections were 12-13 mm long. Root canals were cleaned up to size #40 using step back technique, and filled with gutta-percha and AH26 sealer by the lateral condensation method. Once sealer had set (after 24 hours), the apical 3-mm of root-ends were resected using a high-speed handpiece with a 008 diamond fissure bur and copious water supply, perpendicular to tooth long axis. Using the same instruments, 3 mm deep root-end cavities were prepared 3 mm in depth. Prepared teeth were randomly divided into four experimental groups of 12 each which were filled with one of studied materials: White ProRoot MTA (Densply Tulsa Dental, Tulsa, OK), amalgam (SDI, Victoria, Australia), CEM cement, and Root MTA (Salamifar, Tehran, Iran). All materials were prepared according to their instructions. External surfaces of roots were covered with two layers of nail polish to prevent penetration of materials through dentinal tubules and accessory canals. For greater accuracy, radiographs were taken from the prepared teeth. Coronal gutta-percha was removed using headstrom file and reamer; and this was confirmed radiographically.

***Positive controls:*** The three teeth in this group were not filled after preparation. External surfaces of tooth roots were covered using two layers of nail polish.

***Negative controls:*** The canals and root-ends were filled with sticky wax; in order to prevent bacterial leakage, all external surfaces in addition to sectioned apical parts were covered with two layers of nail polish.

**Figure 1:**
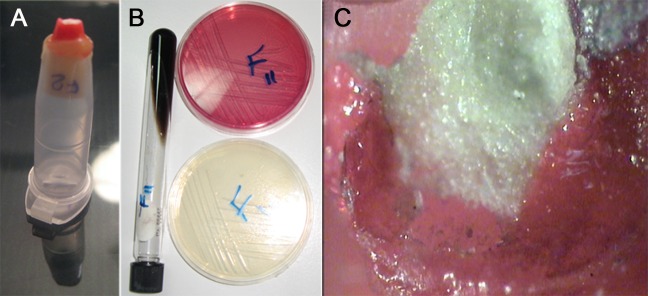
A) The tooth sample was sealed with sticky wax in micropipette; B) blood agar, simple agar, and bile esculin agar containing E. faecalis; C) an apical view of tooth root-end filled with Root MTA (×40)

Double-room technique was used for bacterial leakage measurement [26]. Three-millimeter micropipettes (Supa Co., Tehran, Iran) were used in this study. The 1.5 mm end of pipette was cut and the tooth was inserted from the cap end. The space between tooth and micropipette was sealed with sticky wax (Figure 1A). The micropipettes were placed in sterilized test tubes with identical dimensions to assist a firm fit into the test tube.

### Sterilization:

Fifty-two test tubes each containing 10 mL of Trypticase Soy Broth (TSB) were autoclaved and incubated in 37˚C. The clearness of the media confirmed sterilization. The teeth inside the test tubes were sterilized using ethylene oxide for 12 hours. Under the sterile hood and heat, the micropipettes were inserted into TSB-filled test tubes, and were then isolated using Parafilm (Supa Co., Tehran, Iran). This set was incubated in 37˚C. Clearness of tubes indicated sterilization of samples.

### Bacteria preparation and inoculation:

Enterococcus (E) faecalis were taken from Pasteur Institute of Iran.

For accuracy of bacterial purity, they were cultured in several specific media and then assessed in blood agar, simple agar, and bile esculin agar. Bacteria colonies are uniform and unicolor in blood agar; they show slight bacteria growth in simple agar and black discoloration of media in bile esculin (Figure 1B). Pure bacteria colony derived from blood agar were cultured in 10 cc TSB and were then incubated in 37˚C for 24 hours so that a 0.5 McFarland (1.5×10^8^ bacteria/mL) solution concentration was achieved. All samples were prepared with the same concentration and bacteria induction. Samples were incubated in 37˚C for three days; no turbidity was detected and 0.1 mL bacteria were inserted every five days. Micropipettes and 50 mm of test tubes were sealed using Parafilm (Supa Co., Tehran, Iran). The samples were placed again in an incubator. Opaque samples were excluded from the study. After 70 days, the samples were rinsed with Chlorhexidine and distilled water.

They were then dried, and methylene blue 1% dye was placed on the upper area of retrofilling material. After 72 hours, dye penetration between root filling material and tooth walls was investigated from the apical view with stereomicroscope ×40 (Olympus, Japan) (Figure 1C).

Observation of dye leakage at the interface of root filling material and the tooth root from the apical aspect was considered as complete leakage. Bacterial and dye leakages were statistically analyzed using Fisher’s Exact test.

## RESULTS

**Figure 2:**
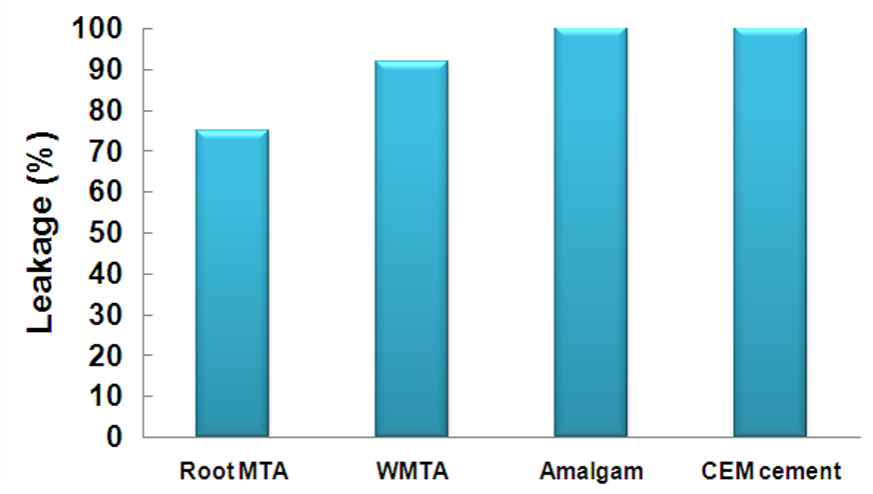
Bacterial leakage in studied groups (after 70 days)

The results of bacterial leakage after 70 days showed complete leakage within 3 days in the positive control samples. Negative samples showed no leakage after 70 days. In the Root MTA group, 9 samples (75%) showed leakage and three samples did not. In WMTA group, 11 samples (91.7%) demonstrated leakage; only one sample did not have leakage. All the samples in amalgam and CEM cement groups demonstrated leakage (Figure 2).

The difference in bacterial leakage of studied materials was not statistically significant.

### Dye leakage:

All positive controls showed complete dye leakage, while negative control group did not demonstrated leakage.

**Figure 3:**
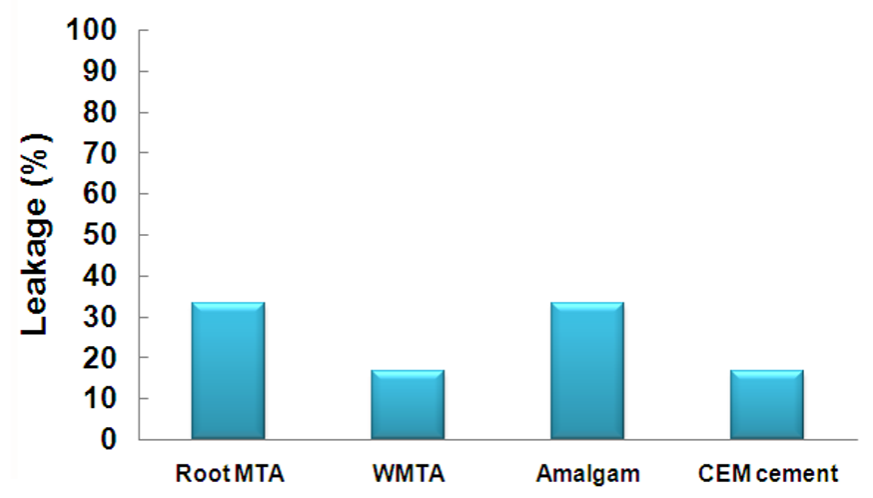
Dye leakage in studied groups (after 3 days)

CEM cement and WMTA groups each had two samples (16.7%) with dye leakage. Four samples (33.3%) of amalgam and Root MTA groups showed dye leakage (Figure 3). The differences between groups were not significant.

### Agreement in leakage measurement with dye and bacteria:

Inter-rater agreement was used to compare the leakage measurement using dye and bacteria methods. Kappa=0.00 was considered as poor agreement [27].

The overall agreement was 29.1% between bacterial leakage and dye leakage.

Dye leakage as a predictor for bacterial leakage had a positive predictive value of 91.7% and a negative predictive value of 8.3%. The sensitivity was 25% and the specificity was 75%.

## DISCUSSION

Dye and bacterial leakage was investigated in this study to assess the efficacy of 4 different root-end filling materials. Dye leakage method is frequently used; however, this method is limited to assessing dye leakage from the root surfaces; i.e. evaluating the material adaptation with cavity walls and the circumferential seal. Moreover, only one section of tooth root will be available for evaluation, which is midsagittal root section. Although bacterial leakage method measures the exact penetration of bacteria, this cannot mirror the clinical condition as only one type of bacteria is used. Also, in this measurement single bacterial penetration demonstrates positive result, but a determined number of bacteria with specific characteristics are needed to cause dental disease. E. faecalis was used in this study as the predominant bacteria in chronic apical periodontitis of failed root canal therapies [11][12]. This study was carried out during 70 days, in accordance with Torabinejad’s study [28]. The same teeth underwent dye leakage (methylene blue 1% after 72 hours) and bacterial leakage tests, i.e. tests were carried out under the same conditions. Using two leakage measurements in this study provided greater information as well as a good comparison between two methods. Also, the microleakage in CEM cement as a new root-end filling material was investigated.

### A) Bacterial leakage

This study showed that after 70 days, bacterial leakage was 75%, 91.7% for Root MTA and WMTA, respectively and 100% for amalgam and CEM cement. The difference was not significant. Asnaashari et al. studied microleakage of Staphylococcus (S) epidermidis in Pro Root MTA, Root MTA and Portland cement and found 33.3%, 40%, and 40% leakage, respectively[29]. The difference between the results may be due to the different bacteria used. E. faecalis which was used in this study is not a dermal or respiratory flora [30][31][32]; this significantly reduces the possibility of errors. Also, our study was performed over 70 days; more than twice than that of Asnaashari. In addition, our study demonstrated significant difference in bacterial leakage between WMTA and Root MTA.

Torabinejad et al. studied the leakage of S. epidermidis during 90 days in MTA, IRM, Super EBA, and amalgam. MTA had the best results [33], concurring with our study; however, they had demonstrated 0% leakage for MTA. This difference may be due to different studied bacteria and study designs. No other study claims 0% leakage for MTA.

Mozayeni et al. investigated S. epidermidis leakage during 60 days in cold ceramic, amalgam, and MTA and showed the least microleakage for MTA [8]; concurring with our results.

Zarrabian et al. demonstrated 50% microleakage for GMTA and Portland cement and 56% for Root MTA over 60 days [29]. The difference between WMTA and Root MTA was not significant.

Fischer et al. investigated leakage of Serratia Marcescens in amalgam, IRM, Super EBA, and MTA. They found that the leakage in MTA was significantly less in MTA [34]. In this study MTA showed less leakage than amalgam; statistically significance was not demonstrated.

### B) Dye leakage 

After 72 hours, microleakage of methylene blue 1% showed 16.7% complete dye penetration in CEM cement and WMTA, and 33.3% in amalgam and Root MTA. The difference was not statistically significant. J. Aqrabawi studied dye leakage in three different root-end filling materials including Super EBA, MTA and amalgam. MTA did not showed complete dye leakage while 56% of samples in amalgam group demonstrated complete dye leakage [35]; agreeing with our results. Torabinejad also showed that MTA causes significantly less microleakage when compared to IRM, Super EBA, and amalgam [36].

### C) Inter-rater agreement between bacteria and dye leakage

The agreement between two methods was poor and no significant agreement was achieved between the results of the two methods (P=1.00). In this study the dye was placed over root-end filling material so that gravity can assist the process; however complete penetration was less in the dye compared to bacterial method. According to Barthel, this can be explained by the different factors which are influenced by bacteria (ion charge, pH, and temperature) but cannot be restored with dye. In addition, there are other factors which can support bacterial penetration i.e. bacteria deformation, active movement, mitosis and growth. Martell investigated dye and electrical leakage and showed different results [37]. Pommel et al. studied three microleakage methods including dye leakage, fluid filtration, and electrochemical which showed different results [38]. Wu et al. stated that after fluid transport for 3 hours under low pressure, dye leakage increased; this was claimed to be due to elimination of entrapped air [39].

Poor agreement between the two used methods has been shown by Barthel et al. [14] adding further weight to the argument that dye leakage cannot predict bacterial leakage. One probable reason may be due to sealing of the leakage pathways via colonies of E. faecalis. Leakage studies cannot be repeated; therefore, extrapolating these ex vivo results for clinical use is uncertain. This is clearly illustrated by the cold lateral condensation technique which causes great leakage in vitro, yet has a 90% clinical success rate [36].

## CONCLUSION

There is a poor agreement between dye and bacterial leakage; as a result, bacterial leakage cannot be envisaged according to dye leakage results. In addition, no significant difference was detected between different groups; consequently it can be concluded that CEM cement provides comparable leakage results with other used rootend filling materials such as MTA.
